# Comprehensive variant calling from whole‐genome sequencing identifies a complex inversion that disrupts *ZFPM2* in familial congenital diaphragmatic hernia

**DOI:** 10.1002/mgg3.1888

**Published:** 2022-02-04

**Authors:** Thomas J. Nicholas, Najla Al‐Sweel, Andrew Farrell, Rong Mao, Pinar Bayrak‐Toydemir, Christine E. Miller, Dawn Bentley, Rachel Palmquist, Barry Moore, Edgar J. Hernandez, Michael J. Cormier, Eric Fredrickson, Katherine Noble, Shawn Rynearson, Carson Holt, Mary Anne Karren, Joshua L. Bonkowsky, Martin Tristani‐Firouzi, Mark Yandell, Gabor Marth, Aaron R. Quinlan, Luca Brunelli, Reha M. Toydemir, Brian J. Shayota, John C. Carey, Steven E. Boyden, Sabrina Malone Jenkins

**Affiliations:** ^1^ Department of Human Genetics, Utah Center for Genetic Discovery University of Utah Salt Lake City USA; ^2^ ARUP Laboratories Salt Lake City USA; ^3^ Department of Pathology University of Utah Salt Lake City USA; ^4^ Division of Neonatology, Department of Pediatrics University of Utah School of Medicine Salt Lake City USA; ^5^ Division of Pediatric Neurology, Department of Pediatrics University of Utah School of Medicine Salt Lake City USA; ^6^ Primary Children's Center for Personalized Medicine Salt Lake City USA; ^7^ Division of Pediatric Cardiology, Department of Pediatrics University of Utah School of Medicine Salt Lake City USA; ^8^ Department of Biomedical Informatics University of Utah Salt Lake City USA; ^9^ Division of Medical Genetics, Department of Pediatrics University of Utah School of Medicine Salt Lake City USA

## Abstract

**Background:**

Genetic disorders contribute to significant morbidity and mortality in critically ill newborns. Despite advances in genome sequencing technologies, a majority of neonatal cases remain unsolved. Complex structural variants (SVs) often elude conventional genome sequencing variant calling pipelines and will explain a portion of these unsolved cases.

**Methods:**

As part of the Utah NeoSeq project, we used a research‐based, rapid whole‐genome sequencing (WGS) protocol to investigate the genomic etiology for a newborn with a left‐sided congenital diaphragmatic hernia (CDH) and cardiac malformations, whose mother also had a history of CDH and atrial septal defect.

**Results:**

Using both a novel, alignment‐free and traditional alignment‐based variant callers, we identified a maternally inherited complex SV on chromosome 8, consisting of an inversion flanked by deletions. This complex inversion, further confirmed using orthogonal molecular techniques, disrupts the *ZFPM2* gene, which is associated with both CDH and various congenital heart defects.

**Conclusions:**

Our results demonstrate that complex structural events, which often are unidentifiable or not reported by clinically validated testing procedures, can be discovered and accurately characterized with conventional, short‐read sequencing and underscore the utility of WGS as a first‐line diagnostic tool.

## Graphical abstract

As part of the Utah NeoSeq project, we used a research‐based, rapid whole‐genome sequencing (WGS) protocol to investigate the genomic etiology for a newborn with a left‐sided congenital diaphragmatic hernia (CDH) and cardiac malformations, whose mother also had a history of CDH and atrial septal defect. Using both a novel, alignment‐free and traditional alignment‐based variant callers, we identified a maternally‐inherited complex SV on chromosome 8, consisting of an inversion flanked by deletions. This complex inversion, further confirmed using orthogonal molecular techniques, disrupts the ZFPM2 gene, which is associated with both CDH and various congenital heart defects. Our results demonstrate that complex structural events, which often are unidentifiable or not reported by clinically validated testing procedures, can be discovered and accurately characterized with conventional, short‐read sequencing and underscore the utility of WGS as a first‐line diagnostic tool.

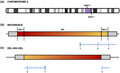

## 1 INTRODUCTION

For many critically ill infants, genome sequencing provides a promising method for ascertaining the cause of a wide range of rare disease phenotypes. Furthermore, rapid genetic diagnosis has enabled therapeutic interventions that ameliorate or resolve some clinical conditions (Chen et al., 2018; Chen et al., 2018; Owen et al., 2021; Sanford et al., 2019; Saunders et al., 2012). Despite substantial advances in DNA sequencing technologies and analytical tools, the majority of cases yield no diagnostic variants and the etiology of these infants remains unresolved. While some of these unsolved cases may lack a genetic explanation, others are likely missed because certain types of genetic variation are more difficult to identify and validate (Mahmoud et al., 2019). Complex structural variants (SVs) involving combinations of deletions, duplications, and copy‐neutral rearrangements of genomic content are particularly difficult to identify given the diversity of sequencing signals they may produce when compared to a reference genome. Because of these challenges, many complex structural events remain uncharacterized and their functional impacts are thus poorly understood. However, complex SVs can disrupt large genomic regions and have the potential to cause significant phenotypic consequences (Eichler, 2019). Long‐read sequencing offers a potential improvement towards better identification of complex SVs (Sanchis‐Juan et al., 2018; Wahlster et al., 2021), but is not widely utilized clinically because of high costs and limited throughput. Methods that can detect complex events from commonly available short‐read data will enhance current clinical sequencing analysis efforts and improve the diagnostic yield of these already widely used sequencing methods.

Here we show that comprehensive genetic variant detection, analysis and interpretation identified a complex SV consisting of a large inversion flanked by a pair of deletions (“DEL‐INV‐DEL”) in an infant and her mother, both with congenital diaphragmatic hernia (CDH). This SV was discovered as part of the Utah NeoSeq project, which has designed and implemented a research‐based, rapid whole‐genome sequencing (WGS) protocol to provide genetic diagnoses for critically ill infants in the University of Utah Hospital neonatal intensive care unit (NICU). We further validated the DEL‐INV‐DEL with orthogonal molecular methods. Although our results are derived from a case of familial CDH, they demonstrate the general utility of standard short‐read WGS, accompanied by thorough variant calling and analysis, in determining genetic diagnoses for any type of birth defect or rare congenital disease.

## 2 METHODS

### 2.1 Sample preparation and sequencing

All subjects were enrolled in the Utah NeoSeq study under IRB‐approved methods and guidelines for Human Subjects research. Umbilical cord blood from the proband and parental venous blood samples were obtained and used for DNA sequencing and analysis. Genomic DNA was extracted from 1 mL whole blood using the Chemagic Magnetic Separation Module I (PerkinElmer, MA, USA). DNA was quantified with a broad‐range double‐stranded assay kit on a Qubit 2.0 fluorometer (ThermoFisher Scientific). Dual‐indexed, paired‐end, whole‐genome libraries were prepared from 500 ng input DNA using the standard Illumina DNA Prep workflow (Illumina). Briefly, this protocol uses on‐bead transposomes to normalize the DNA fragmentation and adapter ligation process. The normalized product underwent five PCR cycles to add unique 10‐bp dual indices and sequencing adapters, and double‐sided bead selection was used to select appropriately‐sized fragments. Libraries were quantified via electrophoresis on the 4200 TapeStation with high sensitivity D5000 tapes (Agilent), diluted to 3 nM, and pooled at equimolar ratios. The final library pool was further diluted to 1.6 nM and spiked with 1% PhiX bacteriophage DNA as a sequencing control (Illumina). This pool was denatured and loaded on a NovaSeq 6000 instrument for 2 × 150 bp paired‐end sequencing on an S1 flow cell (Illumina). Sequence reads were generated using bcl2fastq (v2.20) and were demultiplexed and securely transferred using a custom script.

### 2.2 Alignment and SNV/INDEL calling pipeline

The Utah NeoSeq pipeline performs alt‐aware alignment and variant calling against the GRCh38 build of the human reference genome with alt and decoy contigs. The pipeline includes FastQForward, an in‐house multiprocess‐parallelization manager that wraps BWA‐MEM (Li, 2013) for alignment, SAMBLASTER (Faust & Hall, 2014) for duplicate read marking, Sambamba (Tarasov et al., 2015) for data manipulation, and Sentieon Haplotyper and GVCFtyper for variant identification and joint genotyping, as adapted from GATK best practices (Van der Auwera et al., 2013). VEP (McLaren et al., 2016) was used for variant annotation. Full automation of the NeoSeq pipeline uses NICUWatch (https://github.com/srynobio/NICUWatch), an automated codebase to manage sample data transfer, multi‐platform project creation, workflow implementation in Nextflow (Di Tommaso et al., 2017), and Amazon SNS messaging service to inform team members of data processing status and enable rapid downstream analysis. Data quality control reports were generated from fastp (Chen et al., 2018; Chen et al., 2018), indexcov (Pedersen et al., 2017), Alignstats (https://github.com/jfarek/alignstats), and BCFtools stats (Li, 2011), and were aggregated with MultiQC (Ewels et al., 2016). Peddy (Pedersen & Quinlan, 2017) was used to check ancestry, sex, and family relationships. Phenotype‐specific candidate genes were checked for sufficient sequence depth using seqcover (https://github.com/mikecormier/neoseq‐seqcover‐nf).

### 2.3 Single nucleotide variant and insertion–deletion analysis

Slivar (Pedersen et al., 2021) was used to filter for maternally‐inherited heterozygous SNVs and INDELs with a population ALT allele frequency less than 0.2% in both gnomAD v2 (LiftOver to GRCh38) and gnomAD v3, and classified as high impact (defined according to Slivar's internal ranked list of VEP‐annotated variant consequences and using its default cutoff, which includes all nonsynonymous and splice site variants). We also filtered for high impact de novo variants <0.1% and recessive variants <2%, in case the proband and her mother did not share the same genetic etiology. In parallel, SNVs and INDELs were also prioritized using the statistical approaches of VAAST (Hu et al., 2013; Yandell et al., 2011) and Phevor (Singleton et al., 2014) implemented within Fabric Genomics' GEM framework (Vega et al., 2021).

### 2.4 Short tandem repeat analysis

Short tandem repeat (STR) genotypes were called using gangSTR (Mousavi et al., 2019) and STRling (https://github.com/quinlan‐lab/STRling). Both call sets were intersected with a list of 37 STR loci with known disease associations; gangSTR results were filtered using dumpSTR (Mousavi et al., 2021) and STRling results were filtered using a custom script and locus‐specific pathogenic repeat expansion thresholds.

### 2.5 Reference‐based structural variant analysis

SVs were called using both Smoove (https://github.com/brentp/smoove) and Manta (Chen et al., 2016) as part of the automated variant calling pipeline described above, using the GRCh38 reference genome. Each tool was run with CRAM files from the proband, mother, and father and parameterized as recommended in the tool's documentation (https://github.com/Illumina/manta). Smoove produced a Variant Call Format (VCF) file that contains deletions, duplications, inversions, and unclassified breakends (BNDs), whereas Manta produced a VCF file that contains deletions, duplications, insertions, and BNDs. Both VCF files were annotated by Smoove with overlapping genes (Ensembl GRCh38.p12) and Duphold metrics (Pedersen & Quinlan, 2019). Population SV allele frequency information from CCDG (Abel et al., 2020), gnomAD (Collins et al., 2020), and the 1000 Genomes Project (Byrska‐Bishop et al., 2021) was added to each VCF using SVAFotate (https://github.com/fakedrtom/SVAFotate) with a reciprocal overlap fraction requirement of 0.8. Variants from both Smoove and Manta VCFs were filtered using Slivar to select for events present in the proband and mother, but absent from the father. Furthermore, based on Duphold's flanking fold change (DHFFC) annotation, deletions with a value greater than 0.7 and duplications with a value less than 1.25 were excluded. To identify rare or unique variants, Max_AF and Max_PopMax_AF from SVAFotate, which reflect the maximum population allele frequencies corresponding to overlapping SVs from publicly available datasets, were required to be less than 0.01. All SVs passing these filters were then reviewed manually, prioritizing those that overlapped genic regions.

### 2.6 Reference‐Independent variant calling with RUFUS

RUFUS (Ostrander et al., 2018; Richter et al., 2020) is a reference‐free, alignment‐independent variant caller that uses unaligned sequence reads to identify sequence k‐mers present in a test sample and absent from one or more controls, extracts reads containing k‐mers unique to the test sample, performs de novo assembly on those reads, and finally compares the assembled contigs to a reference genome in order to report called variants in VCF format. This process enables RUFUS to identify both SNV/INDELs and SVs of all types and sizes. Three separate RUFUS runs were performed in parallel; de novo variants were identified by comparing the proband to both parents to identify k‐mers unique to the proband, while inherited variants were identified by comparing the proband to each parent separately to identify k‐mers inherited from the other parent. All runs used a default k‐mer size of 25, a k‐mer reference panel from the 1000 Genomes Project data to select for rare k‐mers, and final mapping of variants to the GRCh38 reference genome. All resulting VCF files were annotated with VEP and compound heterozygous variants were identified in any gene with variants inherited from both parents (excluding VEP‐classified MODIFIER variants). Impactful SV events were defined as any SV that intersects exon regions of the reference gene set (Ensembl GRCh38.p12).

### 2.7 Variant confirmation

#### 2.7.1 Chromosome analysis

Chromosome analysis and karyotype generation were performed on metaphase preparations from whole blood using standard procedures (Lawce, 2017).

#### 2.7.2 Fluorescence in situ hybridization

Fluorescence in situ hybridization (FISH) studies using fluorescently labeled bacterial artificial chromosome (BAC) clones (Empire Genomics, Buffalo, NY) were performed on interphase nuclei from the proband and an unrelated healthy control sample. A single red fluorescent BAC (RP11‐118J17) was designed to hybridize within the inverted segment, while two green fluorescent BACs (RP11‐10G10 and RP11‐79F7) were designed to flank the breakpoints of the inversion. Fifty interphase cells were scored for each red‐green probe combination.

#### 2.7.3 PCR and Sanger sequencing

The genomic regions flanking the proximal and distal 3.7 kb deletions were amplified and Sanger sequenced using DNA extracted from whole blood samples obtained from the proband, mother, and father. PCR was performed using M13‐tailed primers (Table S1), Premix D (Lucigen), and AccuStart II Taq DNA Polymerase (Quantabio). Long‐range PCR was performed using AccuStart Long‐Range SuperMix (Quantabio). Bidirectional Sanger DNA sequencing was performed with BigDye Terminator Cycle Sequencing Kit (Life Technologies, Thermo Fisher Scientific) and M13 primers (IDT) and products were electrophoresed on an Applied Biosystems 3730 DNA Analyzer. Data analysis was performed using QIAxcel Advanced System (Qiagen), Sequence Scanner Software (Life Technologies, Thermo Fisher Scientific), and NCBI Blast.

## 3 RESULTS

### 3.1 Clinical presentation

In the Primary Children's Hospital fetal care center, a 21‐year‐old gravida 2 para 0 mother was identified to have a pregnancy complicated by a fetus with a left‐sided CDH involving liver, stomach, and bowel intrusion into the chest cavity. The estimated left lung volume was 25.3%. A fetal echocardiogram showed a hypoplastic‐appearing aortic arch, possible coarctation of the aorta, and concern for a hypoplastic left ventricle. Fetal sonogram also noted a urinary tract dilation with intermittent bilateral ureterectasis and a normal bladder.

The pregnancy was complicated by gestational diabetes that was diet‐controlled and preterm, premature rupture of membranes, which required medical induction of labor with cesarean delivery at 34 6/7 weeks gestation. Apgar scores were 1 and 7 at one minute and five minutes respectively. Growth parameters were notable for a small‐for‐gestational‐age infant with weight at the 9th centile, length at the second centile, and head circumference at the 18th centile. Physical examination findings were significant for a scaphoid abdomen, 5th finger distal phalangeal hypoplasia, and positional thumb adduction.

The family history was notable in that the mother was also born with a left‐sided CDH as well as an atrial septal defect (ASD) and scoliosis, both requiring surgical intervention. She had residual pulmonary hypoplasia and pulmonary hypertension following CDH repair. Examination of the mother showed no dysmorphic features of the face. The family history was otherwise unremarkable, with no consanguinity or other relatives with CDH or heart defects.

Echocardiogram on the proband after birth excluded a coarctation but showed a hypoplastic aortic arch and evidence of pulmonary hypertension, as well as a small secundum ASD. The patient underwent CDH repair with muscle flap on day of life 3, with abdominal closure 5 days later. Examination of the craniofacial features showed no notable findings or dysmorphic features of the face or limbs. At 4 months of age the patient underwent a second repair of a recurrent CDH followed by tracheostomy and gastrostomy tube at 6 months of age. The patient's course has been complicated by chylothorax, inferior vena cava thrombus, and severe pulmonary hypertension managed with multiple therapies. The Human Phenotype Ontology terms assigned for WGS analysis included congenital diaphragmatic hernia, hypoplastic aortic arch, atrial septal defect, pulmonary arterial hypertension, hydronephrosis, respiratory failure, intrauterine growth restriction, and premature rupture of membranes.

### 3.2 Variant identification

Rapid WGS produced >45‐fold median coverage for all three sequenced individuals. SNV/INDELs, SVs, and STRs were analyzed to identify rare, maternally‐inherited coding or splice site variants, with particular focus on genes from a previously‐compiled CDH candidate gene list (Supplementary Materials). Analysis of SNV/INDELs and STRs did not reveal any variants that met filtering criteria and were also consistent with the observed CDH and cardiac phenotypes. However, three variant callers designed to detect SVs—Smoove, Manta, and RUFUS—were used concurrently and all three methods identified a heterozygous, 4.37 Mb complex variant in the proband and mother, which was absent from the father. This genomic rearrangement was localized to chromosome 8q22.3–23.1, where it relocates and reverses the orientation of the 5′ end of the *ZFPM2* gene (OMIM:603693, NCBI Reference Sequence: NC_000008.11, GenBank Assembly: GCF_000001405.39), including its first two coding exons.

**FIGURE 1:**
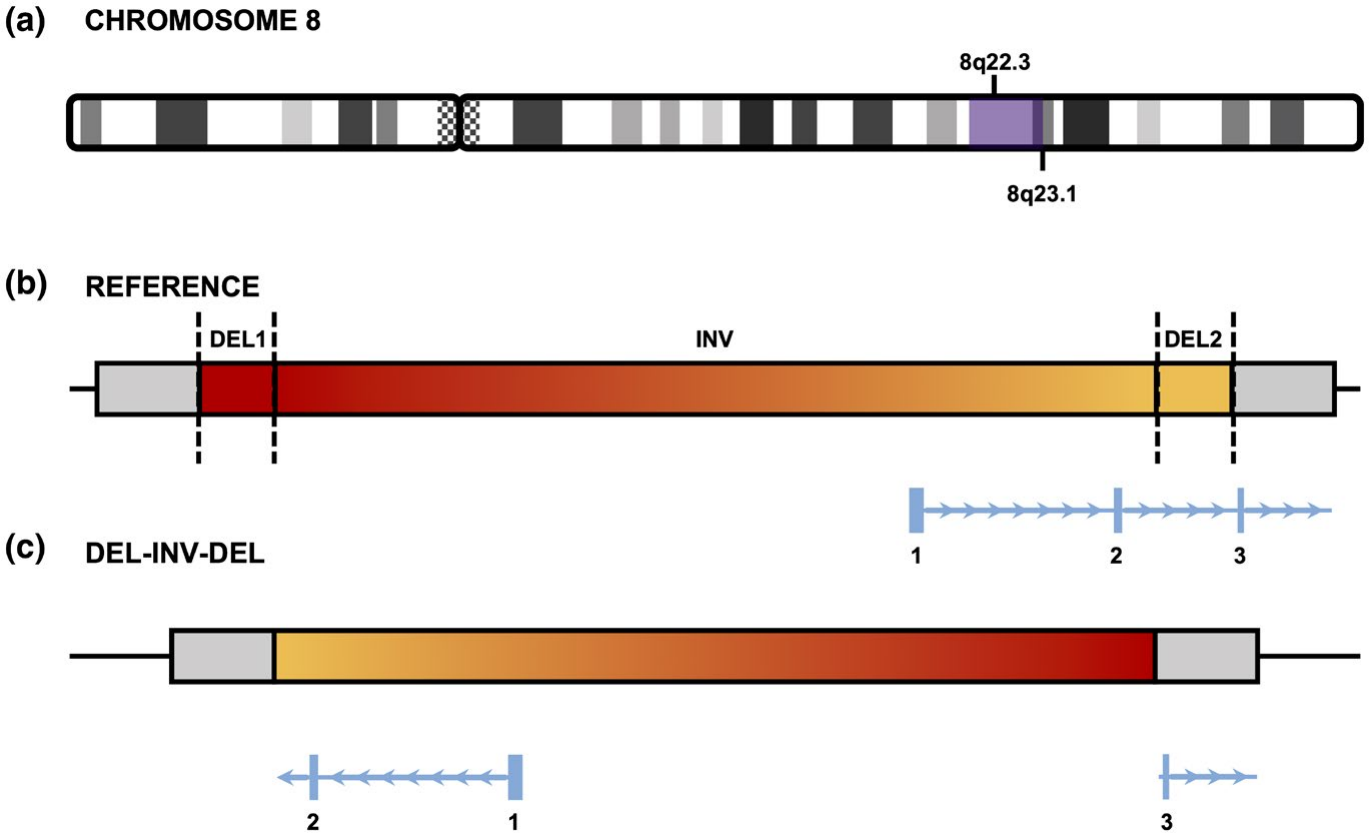
A DEL‐INV‐DEL structural variant disrupts *ZFPM2*. (a) The approximate location of the DEL‐INV‐DEL event is highlighted in purple on the long arm of chromosome 8. (b) The location of the DEL‐INV‐DEL relative to the first 3 exons of *ZFPM2* (in blue). Sizes are not to scale. (c) The expected result of the DEL‐INV‐DEL is to split *ZFPM2* after the second exon, displacing and reversing the orientation of the first and second exons

RUFUS, an alignment‐independent, k‐mer based variant caller, identified a 4.36 Mb inversion and a pair of flanking 3.7 kb deletions as a single, unambiguous, DEL‐INV‐DEL event (Figure S1). Both Smoove and Manta called the same variant as two reciprocal pairs of “breakends” (BNDs). BND variant calls are often omitted from SV analysis because they are a non‐specific variant classification that commonly exhibits a high false positive rate. A BND is called when misoriented or discordant read alignments are detected, but the complete alignment signature does not provide the necessary information for the software to make an automated classification of SV type. As such, a BND call can result from alignment artifacts, but can also be evidence for a translocation, inversion, or more complex structural event. On manual review, the two reciprocal pairs of BNDs generated by Smoove and Manta, when interpreted together, were indicative of a DEL‐INV‐DEL event. Samplot (Belyeu et al., 2021) and IGV (Robinson et al., 2011) were used for visual review of the aligned reads that were informative for Smoove and Manta to call the BNDs, which revealed forward‐forward and reverse‐reverse read pair orientations that are consistent with the breakpoints expected for an inversion (Alkan et al., 2011). Furthermore, discrete drops in read coverage that are often the result of a deletion were observed at the proximal and distal ends of the inversion signal (Figures S2–S4). All together, these observations corroborated that the BND calls from Smoove and Manta represented a complex structural event best described as a heterozygous 4.37 Mb DEL‐INV‐DEL present in the mother and inherited by the proband. The BND calls from Smoove and Manta were consistent with the DEL‐INV‐DEL call from RUFUS with only minor differences (<5 bp) in the location of the breakpoints (Table S2). The inversion entirely encompasses 18 genes and has a 3′ breakpoint in the second intron of *ZFPM2*, which results in the displacement and reversed orientation of the first two exons of this gene (Figure 1). This disruption to the *ZFPM2* gene is expected to cause loss‐of‐function and is interpreted to be “pathogenic” based on PVS1, PM2, and PP1 criteria of the ACMG guidelines (Richards et al., 2015).

### 3.3 Variant confirmation

**FIGURE 2:**
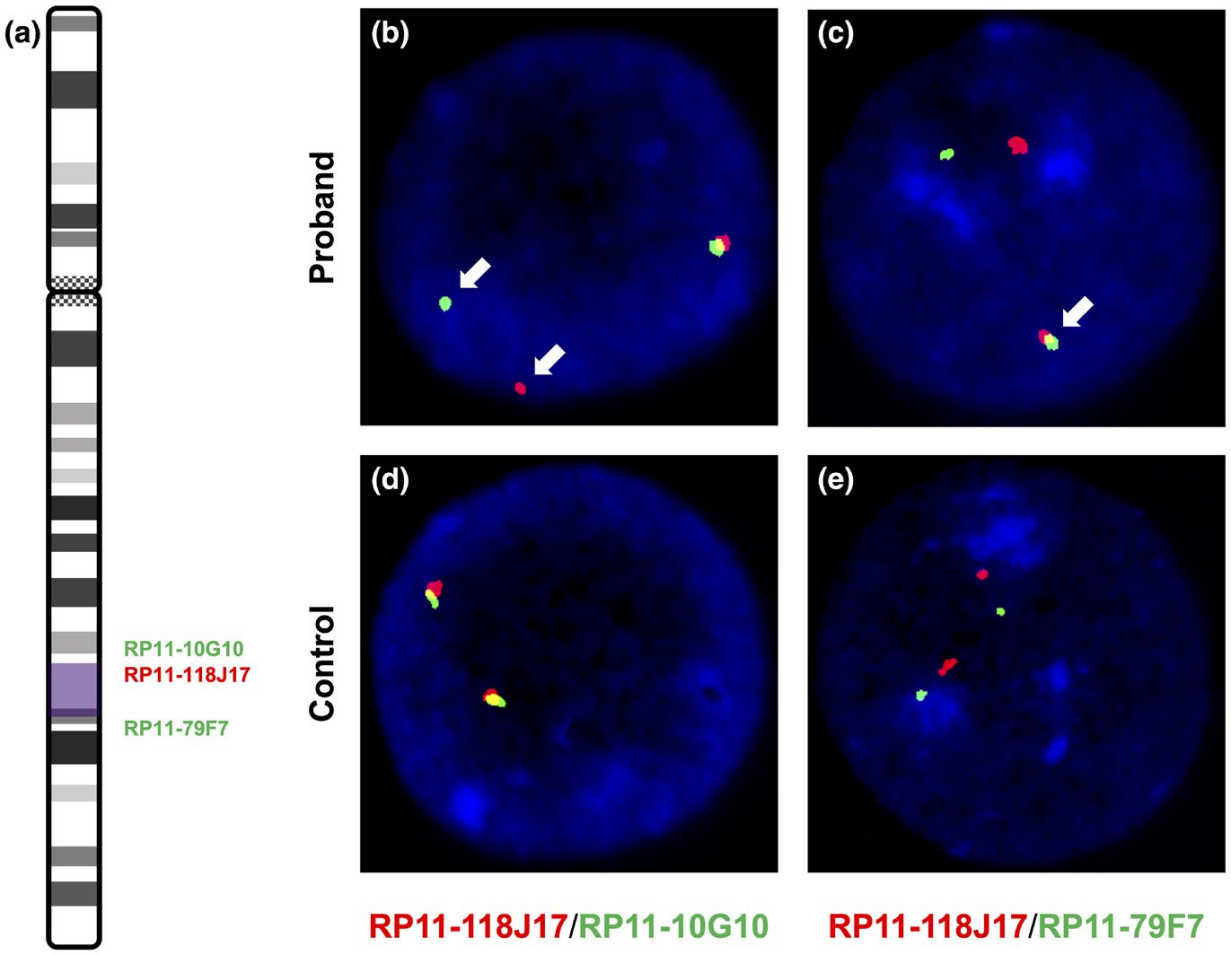
Interphase FISH using fluorescently labeled BAC clone probes. (a) A probe map of the inversion (purple) showing that a single BAC clone was designed within the putatively inverted segment (RP11‐118J17, labeled red), and two clones were designed flanking the inversion (RP11‐10G10, proximal; and RP11‐79F7, distal; both labeled green). The inversion places the RP11‐118J17 probe away from the RP11‐10G10 probe, creating a loss of signal overlap, or “break apart” pattern (b), and brings it closer to the RP11‐79F7 probe, creating a new signal overlap (yellow), or “fusion” pattern (c). The signals highlighting the heterozygous inversion in the proband are indicated with arrows. The signal patterns indicating the lack of an inversion in an unrelated healthy individual for these probe combinations are also shown (d and e). DAPI was used to stain nuclei (blue) of prepared cells

We employed several independent molecular techniques to test for the presence of the DEL‐INV‐DEL. First, G‐banded karyotype analysis of the proband was performed and showed a normal female karyotype (46,XX) (Figure S5). While this analysis ruled out the possibility of a large structural rearrangement not detected by the WGS analysis, the resolution of karyotyping is insufficient to detect the 4.37 Mb event described by WGS. Therefore, interphase FISH analysis using fluorescently labeled BAC clone probes was performed, and two independent experiments produced complementary hybridization patterns that confirmed the presence of the inversion identified as part of the DEL‐INV‐DEL event (Figure 2).

**FIGURE 3:**
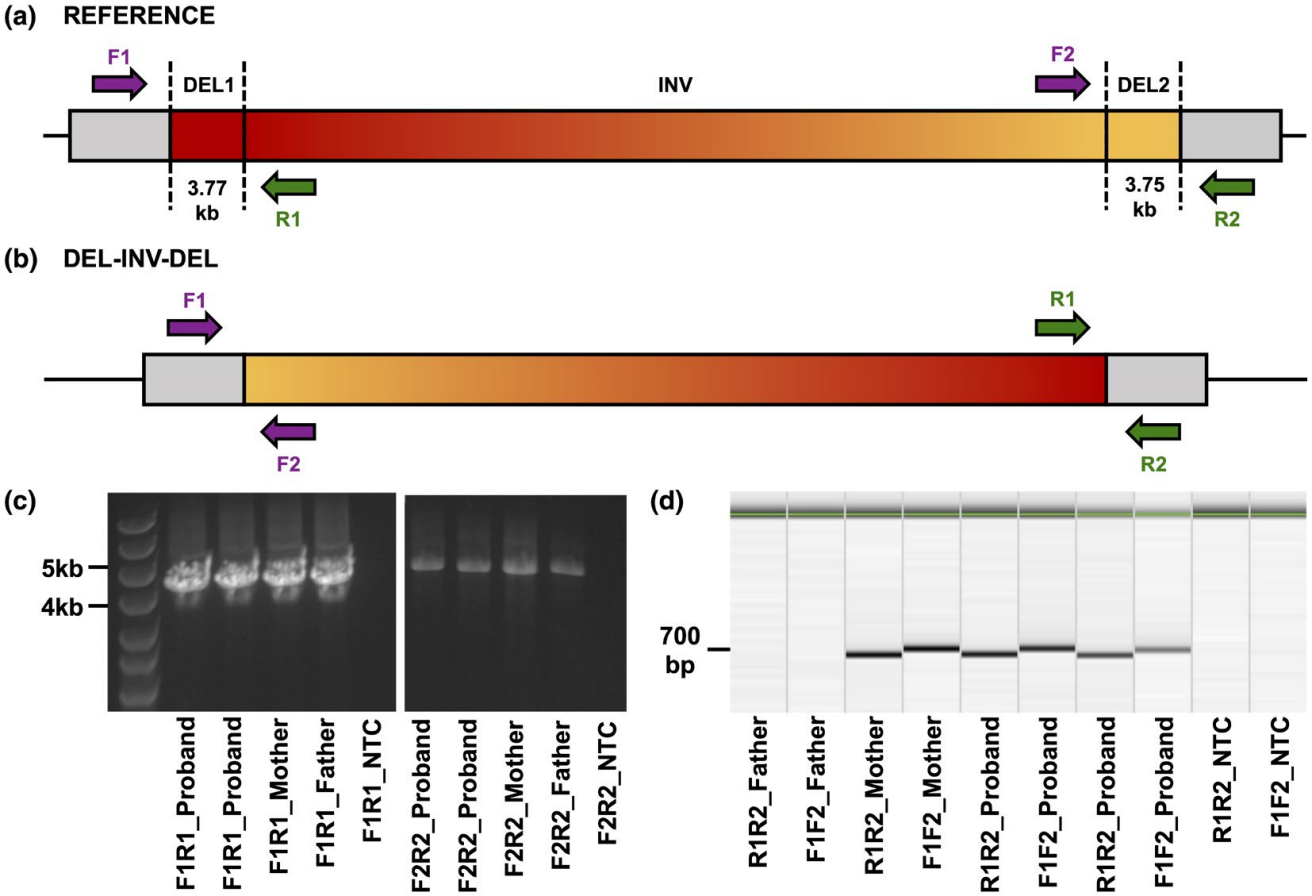
Amplification of the proximal and distal DEL‐INV‐DEL breakpoints. (a). Approximate PCR primer annealing sites flanking the proximal (DEL1) and distal (DEL2) deletions in the absence of the DEL‐INV‐DEL allele, creating F1‐R1 and F2‐R2 primer pairs. (b). Approximate primer annealing sites as a result of the DEL‐INV‐DEL allele, creating F1‐F2 and R1‐R2 primer pairs. (c). Long range amplification of the reference allele using the F1‐R1 and F2‐R2 primer pairs in all three family members (left to right for F1R1 and F2R2: Lane 1: Marker, Lanes 2 and 3: Proband replicates, Lane 4: Mother, Lane 5: Father, Lane 6: Non‐template control; NTC). Amplicons were resolved and sized by standard agarose gel electrophoresis. (d). Amplification of the inverted allele with the R1‐R2 primer pair in the proband (from left to right, Lanes 5 and 7) and mother (Lane 3), but not the father (Lane 1), and amplification with the F1‐F2 primer pair in the proband (Lanes 6 and 8) and mother (Lane 4), but not the father (Lane 2). Amplicons were resolved and sized by electrophoresis using the QIAxcel system (Qiagen)

To confirm the presence of the flanking deletions and further elucidate the breakpoints of the complex DEL‐INV‐DEL, a series of PCR amplifications was conducted. Collectively, the PCR data verify a heterozygous 4.36 Mb chromosomal inversion flanked on both sides by 3.7 kb deletions on chromosome 8q22.3‐8q23.1 in the proband and affected mother, but not the father (Figure 3). The amplicons derived from the inverted segments were further analyzed by Sanger sequencing. As expected, the Sanger reads aligned to the predicted sequence generated using the DEL‐INV‐DEL coordinates from the WGS analysis. Thus, we confirm the exact deletion/inversion breakpoints at the proximal (CAGCAG‐GTATTA; chr8:101077071) and distal (TCCCCT‐ATTAGA; chr8:105442936) ends of this complex variant at nucleotide resolution (Figure S6). These breakpoints were concordant with the RUFUS DEL‐INV‐DEL call and Smoove BND calls, and consistent with the Manta BND calls.

## 4 DISCUSSION

We describe the identification of a complex structural event detected from short‐read, rapid WGS in a family trio, where the SV and the clinical findings of left‐sided CDH and ASD co‐segregated in the mother and infant proband. Using multiple molecular techniques, we confirmed the presence of the variant in the proband and mother, its absence in the father, and its exact breakpoints. This complex SV consists of an inversion flanked by a pair of deletions on chromosome 8, where it reverses and displaces the first two exons of the *ZFPM2* gene. *ZFPM2* is a member of the FOG family of transcription factors, which modulates activity of GATA family proteins. GATA proteins are known regulators of both hematopoiesis (Cantor & Orkin, 2002; Crispino & Horwitz, 2017; Gao et al., 2015) and cardiogenesis (Pikkarainen et al., 2004; Turbendian et al., 2013). Mutations in many GATA family members have resulted in congenital heart defects, including atrial septal defects, and variants in some GATA genes can also cause CDH (Lin et al., 2010; Maitra et al., 2010; Tomita‐Mitchell et al., 2007; Wang et al., 2011; Yu et al., 2013, 2014; Zhang et al., 2008). Heterozygous loss‐of‐function variants in *ZFPM2* have been previously associated with CDH and cardiac malformations (Ackerman et al., 2005; Bleyl et al., 2007; Luca et al., 2011; Tan et al., 2012), establishing haploinsufficiency as the molecular mechanism for pathogenesis. Given the known association between CDH and *ZFPM2*, we conclude that this complex variant explains the CDH and cardiac phenotypes shared by the proband and mother. A significant challenge in the diagnosis and treatment of CDH has been low yields of genetic testing, with a pathogenic genetic change identified in only 30% of cases (Yu et al., 2020). Our results further support recent studies that employ WGS which found a diverse array of mutational types that cause CDH (SNV/INDELs, SVs, and splice site changes) and show the utility of WGS as a first‐line diagnostic tool (Bogenschutz et al., 2020; Sweeney et al., 2018).

Clinical care of critically ill neonates is challenging and is exacerbated in the absence of a definitive diagnosis. WGS can empower the comprehensive, detailed, and affordable genetic diagnosis required for personalized medicine. In turn, rapid genetic diagnosis can reduce morbidity and mortality, facilitate end‐of‐life care decisions when needed, and decrease costs of care in the NICU (Daoud et al., 2016; Petrikin et al., 2018; Saunders et al., 2012; van Diemen et al., 2017; Willig et al., 2015). Here, WGS identified the underlying CDH and cardiac etiology, bringing clarity to the team of care providers. The patient's critically ill status and severe pulmonary hypertension were of ongoing concern when unexplained, but having a genetic cause limited the need for further diagnostic investigation. The parents of this patient were interested in understanding the inherited cause of CDH and benefited from having this information for purposes of future family planning, reproductive care, and risk assessment for other family members.

Complex SVs like the DEL‐INV‐DEL identified here often elude conventional WGS variant calling pipelines (Sedlazeck et al., 2018). While long‐read sequencing may help identify complex SVs (Wahlster et al., 2021), our study demonstrates that even complex genomic rearrangements can be discovered using readily available short‐read technologies. Importantly, we relied on a combination of structural variant callers that each identified the signal for the complex event but reported it in different ways. Two of these SV callers (Smoove and Manta) called the DEL‐INV‐DEL as a set of BNDs, which highlights the importance of considering and resolving this often‐ignored class of SV calls. This work also demonstrates the unique capability of the alignment‐free variant caller RUFUS, which uses a de novo assembly step and fully described the DEL‐INV‐DEL as a single event with nucleotide‐accurate breakpoints.

We further demonstrated that the complex DEL‐INV‐DEL was not identified by G‐banded karyotype analysis, and we infer that it would not be detected by SNP microarray, exome sequencing, or targeted gene panel sequencing. FISH is not practical for genome‐wide screening and is only used clinically for targeted testing. Accordingly, we propose that only comparable WGS‐based approaches would be able to detect this event or similar structural variants. In addition, clinically validated WGS‐based assays may also have failed to detect or report this structural event, since inversions and other copy‐neutral or complex SVs are often not a validated variant type (The NICUSeq Study Group, 2021). Moreover, both flanking deletions were contained entirely within non‐coding sequence, so even if the deletions had been detected by other methodology, they likely would not have been interpreted as pathogenic on their own. Thus, the comprehensive, research‐based Utah NeoSeq protocol was uniquely well‐suited for the successful characterization of a complex pathogenic SV and provides a general diagnostic approach for identifying such events from commonly available short‐read sequencing data.

## CONFLICT OF INTEREST

Mark Yandell and Barry Moore have financial relationships with Fabric Genomics, which provided web‐based analysis tools used in this study.

## Supporting information


Supporting information

## Data Availability

Data sharing not applicable.
