# Supplementary material for: Comprehensive variant calling from whole‐genome sequencing identifies a complex inversion that disrupts ZFPM2 in familial congenital diaphragmatic hernia
Source: Mol Genet Genomic Med. 2022 Feb 4;10(4):e1888. doi: 10.1002/mgg3.1888 (PMC9000945; doi:10.1002/mgg3.1888)
Supplement: Supplementary file 1 — Supporting information [file MGG3-10-e1888-s001.docx]

**CDH Candidate Gene List**

*ARID1A*, *ARID1B*, *CDKN1C*, *CHAT*, *CHD7*, *COL3A1*, *COX7B*, *CTBP1*, *CTBP2*, *DES*, *DLL3*, *DNASE2*, *EFNB1*, *EYA1*, *EYA2*, *FBN1*, *FGFR2*, *FGFRL1*, *FRAS1*, *FREM1*, *FREM2*, *GAB1*, *GATA4*, *GATA6*, *GLI2*, *GLI3*, *GPC3*, *HCCS*, *HDAC8*, *HES7*, *HLX*, *HOXB4*, *ILF3*, *KDM6A*, *KIF7*, *KMT2D*, *LFNG*, *LRP2*, *LTBP4*, *MESP2*, *MET*, *MMP14*, *MMP2*, *MSC*, *MYOD1*, *MYOG*, *MYRF*, *NDUFB11*, *NEDD4*, *NIPBL*, *NR2F2*, *PAX3*, *PAX7*, *PBX1*, *PDGFRA*, *PIGN*, *POGZ*, *PORCN*, *RAD21*, *RARA*, *RARB*, *RIPPLY2*, *RLIM*, *ROBO1*, *ROBO2*, *SIM2*, *SIX1*, *SIX4*, *SLC2A10*, *SLIT3*, *SMARCA4*, *SMARCB1*, *SMARCE1*, *SMC1A*, *SMC3*, *SOX11*, *SOX7*, *STRA6*, *TBX5*, *TBX6*, *TCF21*, *WT1*, *ZFPM2*

**Supplementary Table 1: M13-tailed primer sequences**

| **Primer Name** | **Sequence** |
| --- | --- |
| F1 | TGTAAAACGACGGCCAGTGCCAGACACTGTGATAGGTCC |
| F2 | TGTAAAACGACGGCCAGTTGGCTTCTTCATGGTCAGTCT |
| R1 | CAGGAAACAGCTATGACCGGAATCCGCAACATGCAGTC |
| R2 | CAGGAAACAGCTATGACCGGAGTCTTACTCGCTCTGTCA |
| F2R* | CAGGAAACAGCTATGACCTGGCTTCTTCATGGTCAGTCT |
| R1F** | TGTAAAACGACGGCCAGTGGAATCCGCAACATGCAGTC |

* F2 primer with reverse M13 tail

** R1 primer with forward M13 tail

**Supplementary Table 2: Selected fields from SV VCFs from three software tools**

| **#CHROM** | **POS** | **ID** | **REF** | **ALT** | **INFO** |
| --- | --- | --- | --- | --- | --- |
| **Smoove** |  |  |  |  |  |
| chr8 | 101077071 | 7880_1 | N | N]chr8:105439188] | SVTYPE=BND |
| chr8 | 101080841 | 7881_1 | N | [chr8:105442936[N | SVTYPE=BND |
| chr8 | 105439188 | 7880_2 | N | N]chr8:101077071] | SVTYPE=BND |
| chr8 | 105442936 | 7881_2 | N | [chr8:101080841[N | SVTYPE=BND |
| **Manta** |  |  |  |  |  |
| chr8 | 101077067 | MantaBND:133679 | G | G]chr8:105439188] | SVTYPE=BND |
| chr8 | 101080841 | MantaBND:133638 | A | [chr8:105442937[A | SVTYPE=BND |
| chr8 | 105439184 | MantaBND:133679 | A | A]chr8:101077071] | SVTYPE=BND |
| chr8 | 105442936 | MantaBND:133638 | T | [chr8:101080842[T | SVTYPE=BND |
| **RUFUS** |  |  |  |  |  |
| chr8 | 101077071 | 3769D4358348V3747D | G | <INV> | SVTYPE=INV |

Smoove and Manta called the complex inversion as two pairs of reciprocal breakends (BNDs), whereas RUFUS called it as a single inversion. The ID field from the RUFUS call describes the complex SV explicitly as a 3769 bp deletion (D) followed by a 4358348 bp inversion (V) followed by a 3747 bp deletion (D). INFO fields are truncated to only show the SVTYPE classification, and Manta ID fields are truncated for clarity.


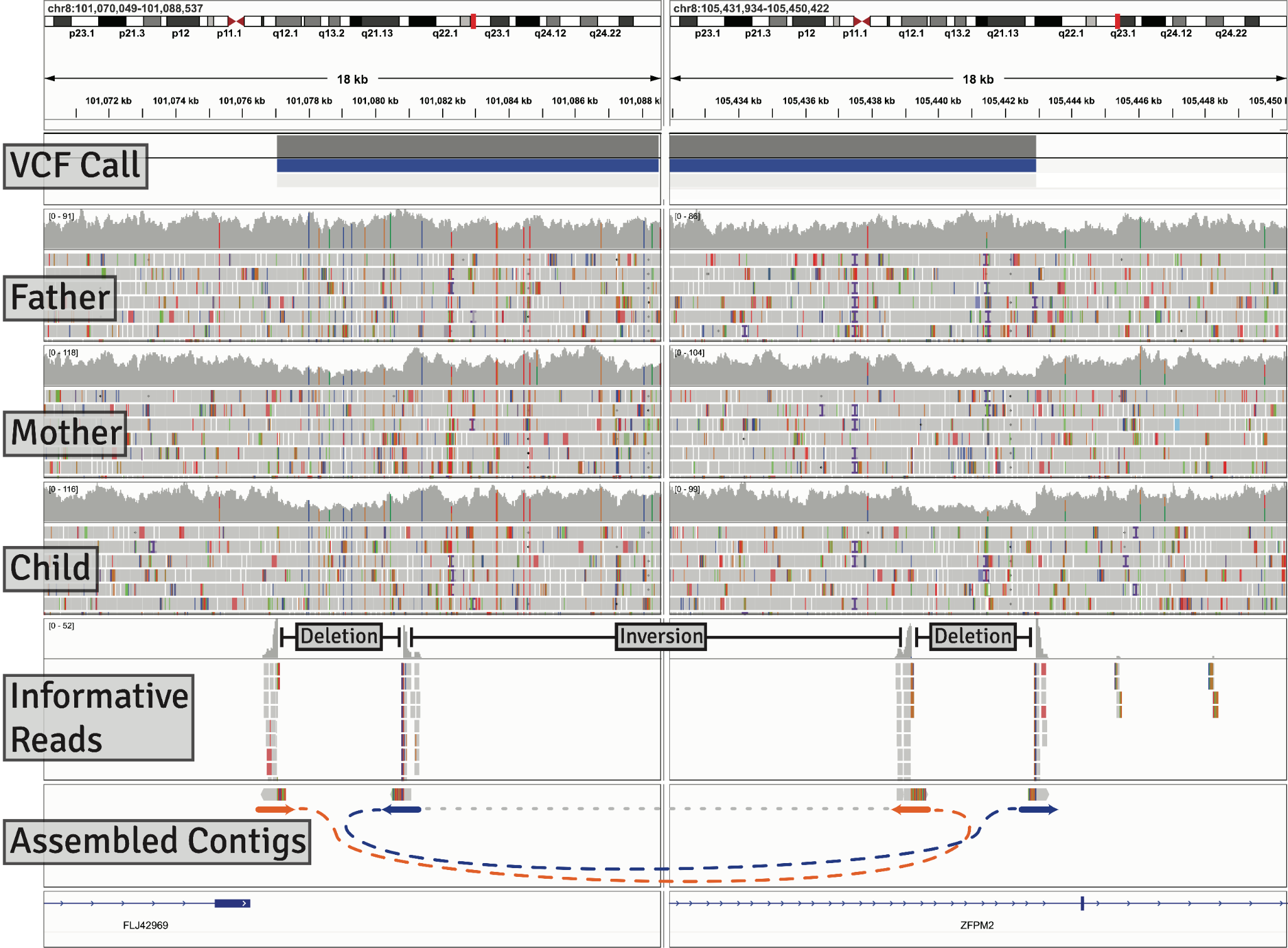


**Supplementary Figure 1: Schematic of the RUFUS Identification of the DEL-INV-DEL Event**

The region corresponding to the DEL-INV-DEL event is pulled from the RUFUS **VCF Call** and highlighted above in blue/gray. Annotated IGV screenshots with tracks **Father**, **Mother**, and **Child** show the full read pileups from the CRAM files for each sample. **Informative Reads** shows the reads RUFUS identified as containing novel kmers present in the child and absent from the unaffected father. These reads were used in downstream analysis including de novo contig assembly, whereas all other uninformative reads from the CRAM files were removed from downstream analysis. **Assembled Contigs** shows the final assembled contigs that describe the breakpoints of the event. Two contigs were identified that describe the DEL-INV-DEL event, depicted as orange (652 bp, 31X depth of coverage) and blue (550 bp, 22X depth of coverage) arrows. Each contig represents a stretch of continuous sequence that is split-mapped when the contig is aligned to the GRCh38 reference, with one resulting piece being reverse oriented. From left to right, the first split orange contig shown aligns to chr8:101,076,648-101,077,071 while the subsequent split orange contig is reverse oriented and maps to chr8:105,438,957-105,439,188 indicating the start on an inversion event. The blue contig then complements the orange contig and describes the resolution of the inversion, where the first split blue contig aligns to chr8:101,080,841-101,081,080 in a reverse orientation and then the remaining split blue contig aligns at position chr8:105,442,936-105,443,246, downstream of the second split orange contig, signaling the return to genomic sequence matching the GRCh38 reference. RUFUS determines the exact breakpoints of the event using these split contig alignments. The distance between the breakpoints of the blue and orange contigs at each inversion breakpoint indicates the size of the deletions. This analysis is automated by RUFUS and is reported as a single event in the VCF with the ID field description 3769D4358348V3747D, indicating a 3769 deletion (D), followed by a 4358348 bp inversion (V), and finally a 3747 deletion (D).


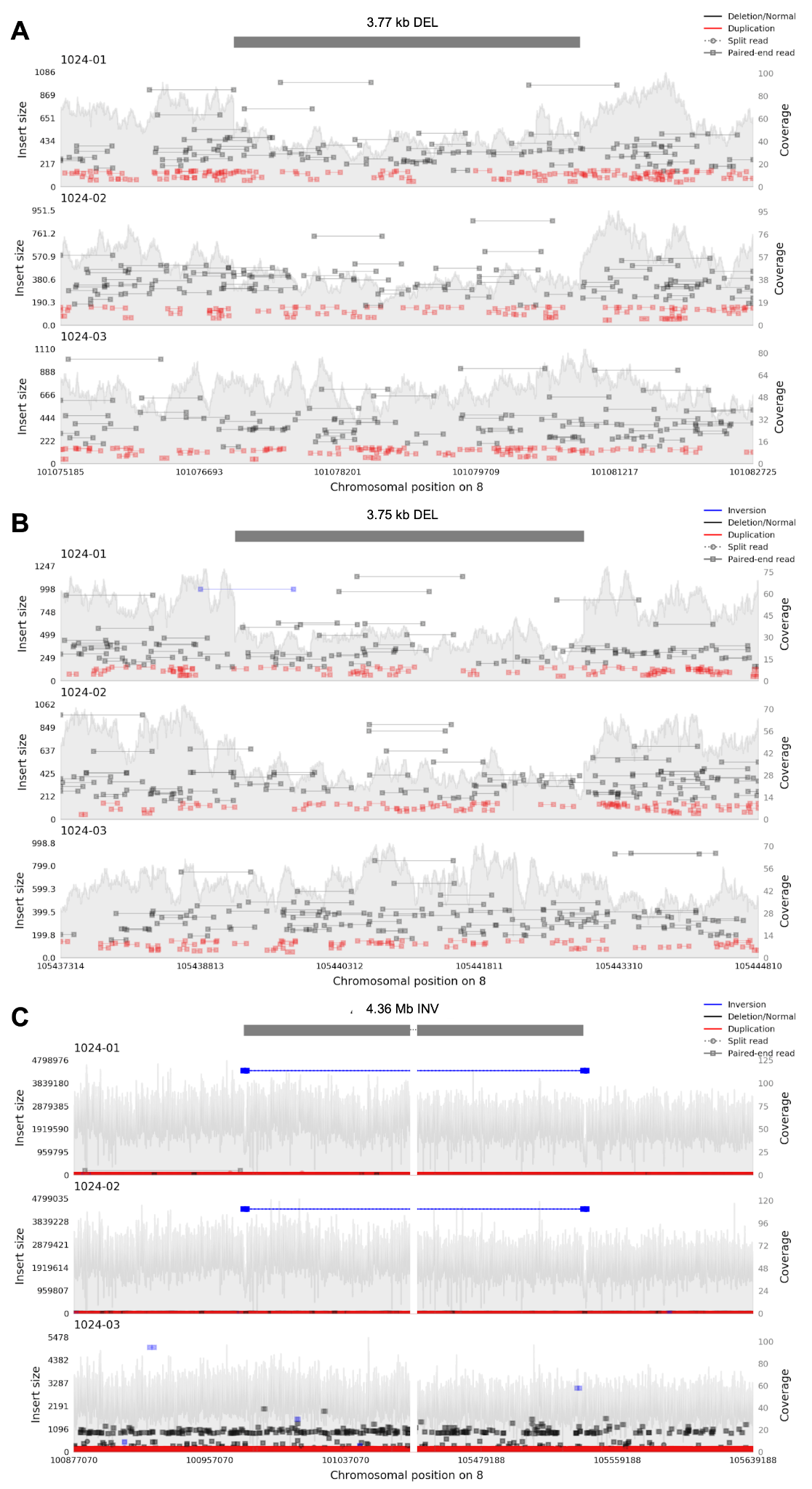


**Supplementary Figure 2. Samplot Images of DEL-INV-DEL**

Samplot images corresponding to the BND calls made by Smoove and Manta. Plotting details are described at <https://github.com/ryanlayer/samplot>. In all images the top plot (K1024-01) refers to the proband, with the middle image (K1024-02) corresponding to the mother and the bottom image (K1024-03) representing the father. **A.** Samplot visualization of the proximal 3.77 kb deletion shows drops in read coverage (in gray) in the proband and mother, but not the father. **B.** Samplot visualization of the distal 3.75 kb deletion also shows drops in read coverage in the proband and mother, but not the father. **C.** Condensed samplot visualization of the 4.36 Mb inversion highlighting misoriented forward-forward and reverse-reverse read-pairs (colored in blue), suggestive of an inversion signal in the proband and mother, but not observed in the father.


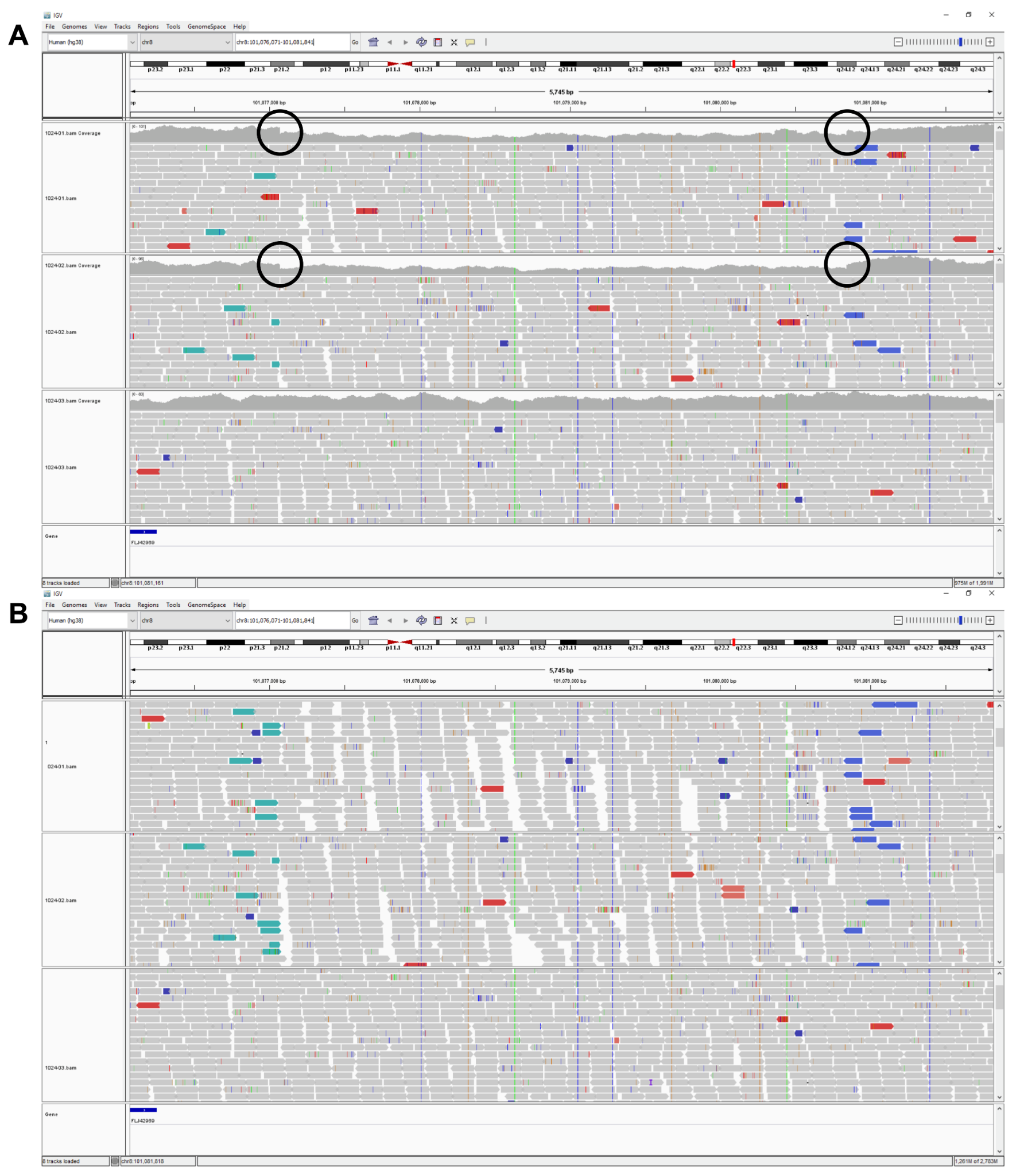


**Supplementary Figure 3. IGV Screenshots of Proximal DEL and INV Breakpoint**

IGV screenshots visualizing the read pileups of the proximal deletion and inversion breakpoint. In both images the samples are listed as proband (K1024-01), mother (K1024-02), and father (K1024-03) from top to bottom. **A.** IGV screenshot of the proximal 3.77 kb deletion with the apparent breakpoints and drops in coverage circled in the proband and mother. **B.** A view corresponding to the same location as **A**, but scrolled down to further highlight the presence of forward-forward (teal) and reverse-reverse (blue) read-pairs, indicative of an inversion in the proband and mother, but absent from the father.

**
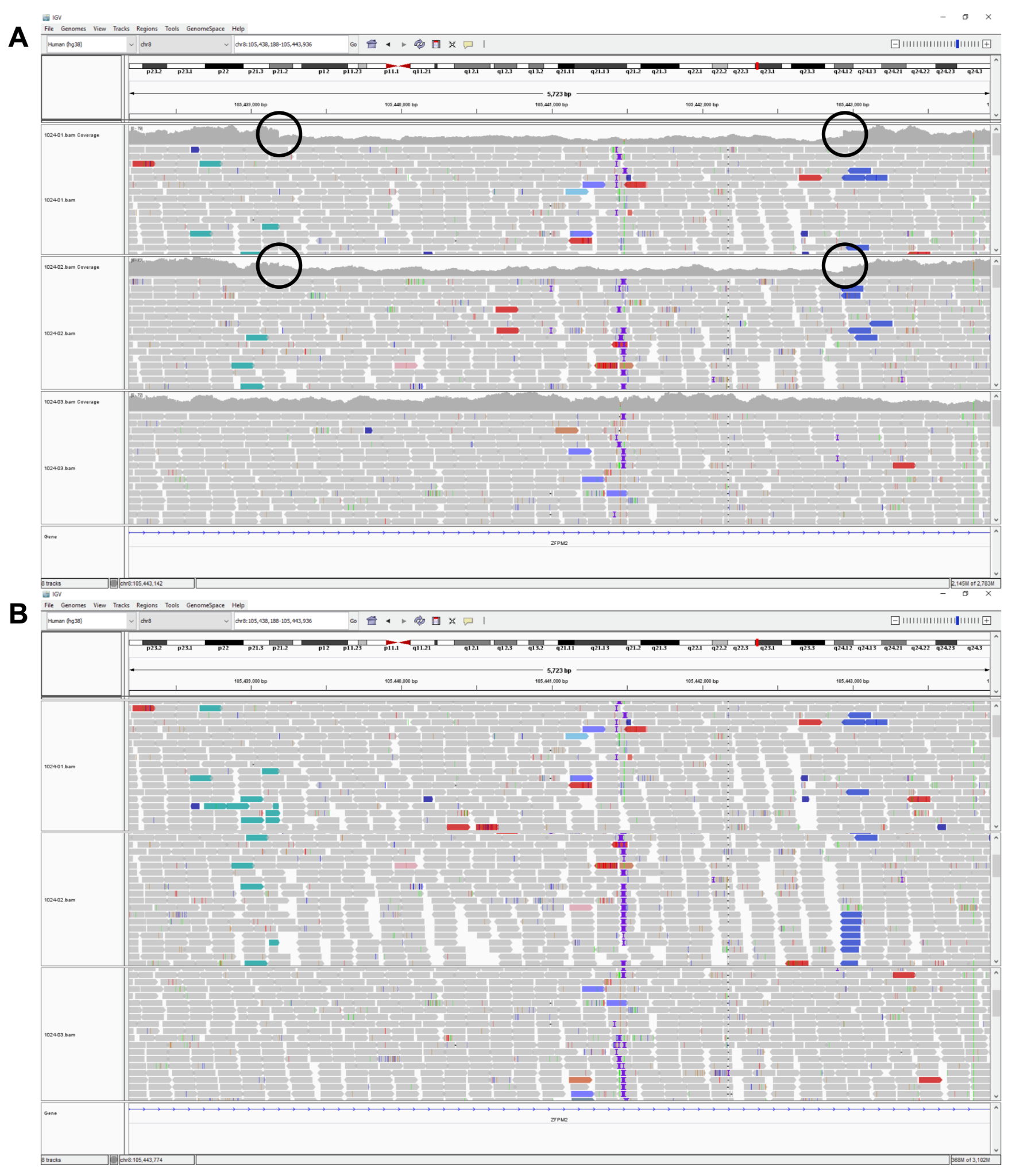
**

**Supplementary Figure 4. IGV Screenshots of Distal DEL and INV Breakpoint**

IGV screenshots visualizing the read pileups of the distal deletion and inversion breakpoint. In both images the samples are listed as proband (K1024-01), mother (K1024-02), and father (K1024-03) from top to bottom. **A.** IGV screenshot of the distal 3.75 kb deletion with the apparent breakpoints and drops in coverage circled in the proband and mother. **B.** A view corresponding to the same location as **A**, but scrolled down to further highlight the presence of forward-forward (teal) and reverse-reverse (blue) read-pairs, indicative of an inversion in the proband and mother, but absent from the father.


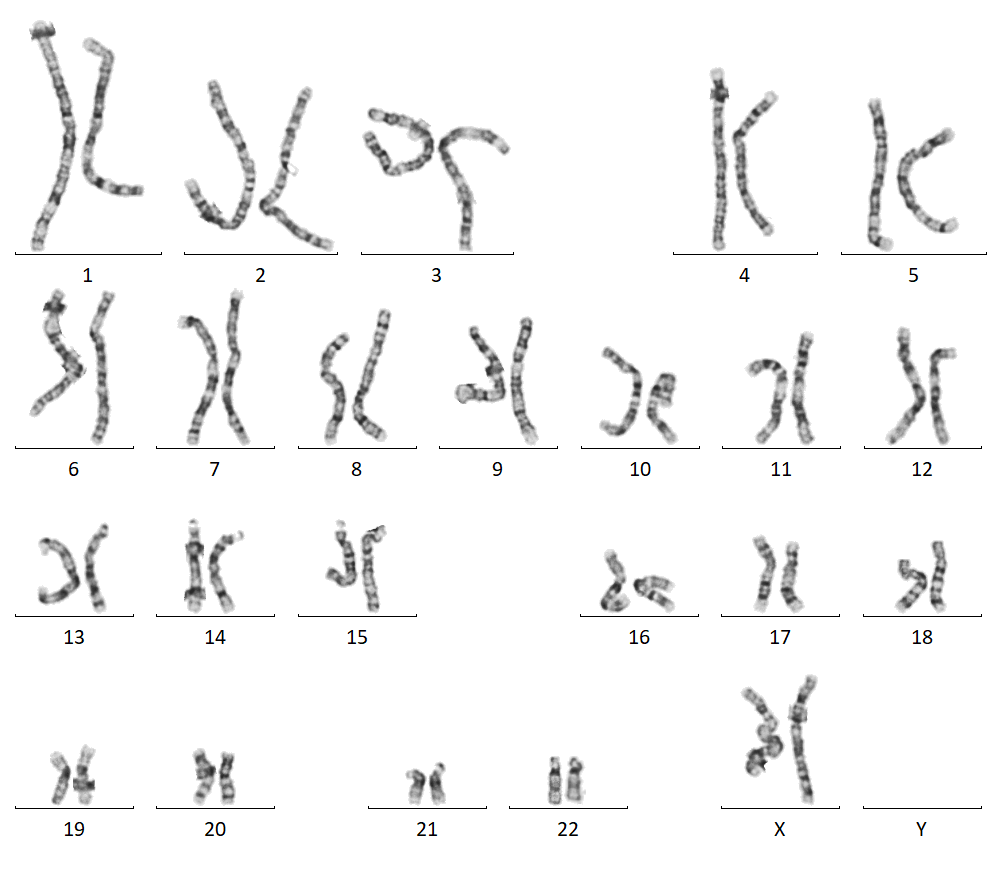


**Supplementary Figure 5: Proband Karyotype**

The proband had a normal, female karyotype with no evident structural change on the long arm of chromosome 8.


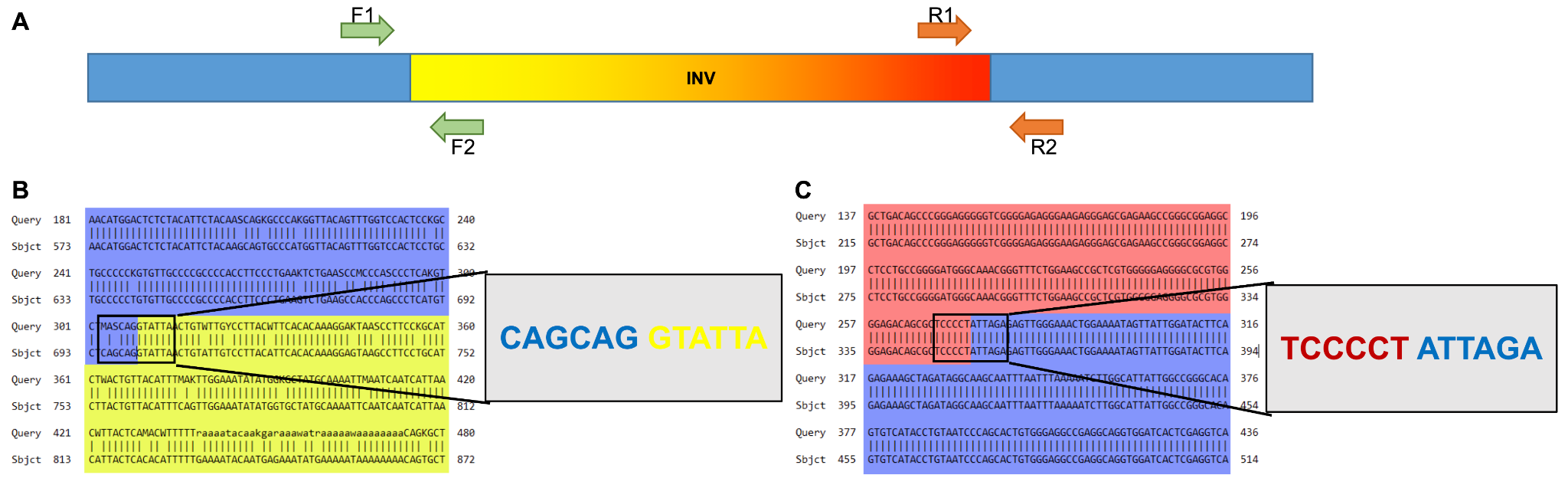


**Supplementary Figure 6: Sanger Sequencing of PCR products resulting from the DEL-INV-DEL variant**

Sanger sequencing of the amplicons derived from the DEL-INV-DEL allele depicted in **(A)**, elucidates at base pair resolution the proximal **(B)** and distal **(C)** deletion-inversion breakpoints for both the proband and mother. Note: sequence is highlighted in blue, yellow, and red in correspondence with **(A)**. fc
